# Factors associated with length of stay in medium secure units: A realist review

**DOI:** 10.1177/10398562241286627

**Published:** 2024-09-24

**Authors:** Wajeeha Zagham, Steve Kisely, Terry Stedman, Karen Brown, Frances Dark

**Affiliations:** The Park, Centre for Mental Health, 157828 West Moreton Hospital and Health Service, Archefield, QLD, Australia; School of Medicine, 1974 The University of Queensland, Woolloongabba, QLD, Australia; The Park, Centre for Mental Health, 157828 West Moreton Hospital and Health Service, Archefield, QLD, Australia; School of Medicine, 1974 The University of Queensland, Woolloongabba, QLD, Australia

**Keywords:** medium secure mental health units, medium secure mental health units, forensic, length of stay, length of stay, realist review

## Abstract

Medium secure mental health units (MSUs) provide a specialised secure environment to assist people recovering from mental illness where less restrictive care has been unsuccessful or been unable to provide safety for the person or the community.

Internationally, it has been found that these units can be associated with prolonged admissions, posing ethical and resource issues.

**Objectives:**

This ‘Realist Review’ aimed to investigate the factors associated with length of stay and outcomes of medium secure care to help inform the development of a local secure care pathway.

**Method:**

The searches generated a total of 1570 entries across multiple search engines. Following removal of duplicates, application of inclusion/exclusion criteria and selection of articles, a total of 18 were reviewed in detail, including a further five articles obtained from references and the explored grey literature.

**Results:**

Several issues influenced not only admission to medium secure units, but also the outcomes. Many articles were retrospective studies relying on administrative data. The realist synthesis provides contextual data to inform program development.

**Conclusions:**

The existing literature, though variable in quality, was limited by the varied jurisdictions and contexts. However it may be useful to inform care pathways for the optimal use of medium secure beds.

Medium secure mental health units (MSUs) exist to provide 24-h secure mental health care for people whose mental health recovery needs are not safely met in other less restrictive forms of care.^1,2^ They may be used as step-down care from more secure forensic mental health facilities and provide a secure mode of care for non-forensic patients who have complex care needs that cannot be met in other parts of the mental health system.^3^

The establishment of forensic mental health services relates back historically to an appreciation in law in the nineteenth century that a person may not be responsible for a crime due to mental illness at the time of the offence (the insanity defence), and were entitled to treatment rather than incarceration. With advances in mental health care treatment, forensic (or secure care) has increasingly incorporated ideas of rehabilitation and a focus on human rights.^4^

Such secure facilities are restrictive, and therefore, there are ethical concerns about who is referred and whether the care achieves the goal of improved patient outcomes. Globally, the ethical issue of unnecessary, prolonged stays in forensic services have been identified.^5–7^

The ideal upper limit for length of admission to medium secure units (MSU) has been defined as 2 years^2^ (Simpson et al., 1976^2^). In 2011, a UK paper identified an increasing trend for patients to be detained in medium secure units for longer than 2 years.^7^ Shah et al. (2011)^7^ cited research identifying an increasing trend for patients in MSUs to be detained beyond 2 years.

A systematic review and meta-analysis conducted in 2016 examined adverse outcomes for patients in secure hospitals from 10 countries and found some evidence for discharged forensic patients having lower offending outcomes, however noted premature mortality, particularly suicide post discharge.^8^ Understanding the optimal use of medium secure beds requires an exploration of care pathways both to, within and from MSUs.^9,10^

This review aims to synthesise the literature on factors associated with length of stay and outcomes of medium secure care to inform referral and care pathways particularly in the Australian context and in the state of Queensland.

Models of service (MOS) for the various service units within Queensland (QLD) mental health service were developed and included medium secure units. The intent behind these MOS was to inform care based on the principles outlined in the Queensland Mental Health Plan.^11^ The Queensland medium secure MOS is not operationally prescriptive about referral pathways or about length of stay. The MOS remains under review. This review was conducted in the context of building a new medium secure unit in Queensland.

## Aim

1) To define factors associated with admission to medium secure units.
2) To determine the factors leading to admissions that are prolonged or exceeding the recommended duration of care.

## Method

A scoping review was undertaken to map the existing evidence related to the topic.^12^ During this phase it became evident that a limited amount of data was available, with only one systematic review obtained.^8^ A realist review was deemed the most appropriate methodology as we were interested in a complex system (medium secure mental health care) where contextual factors are influential in the function of this form of care. This approach is recommended to expand the knowledge base in areas of policy development and for research questions involving ‘what works, for whom, under what circumstances’ and the mechanism and reasons for effect.

Realist reviews can focus on the mechanism of how an intervention, in this case medium secure care works and then inform further improvement in models of the intervention. This form of review aligned to the local context of planned review of the medium secure MOS which applies to Brisbane, QLD.

The methodology and review process as outlined by Pawson^13^ (Table 1) was followed.

**Table 1.** Key steps in realist review (Pawson et al., 2005)^13^

Step 1 Clarify the scope
a.Identify the review question
b.Refine the purpose of the review
c.Articulate key theories to be explored
Step 2. Search for evidence
a.Exploratory background search
b.Refining key theories and inclusion criteria
c.Purposive sampling to test subset of theories with additional snowballing to explore any new hypothesis emerging
d.Final search for additional studies
Step 3. Appraise primary studies and extract data
Step 4. Synthesise evidence and draw conclusions
Step 5 Disseminate

A realist review method was used to compare and analyse acceptable study standards. Quality and consistency was addressed by utilisation of RAMESES (Realist And Meta-Narrative Evidence Syntheses – Evolving Standards) standards^14^ which and the STROBE^15^ checklists which were comparable to Preferred Reporting Items for Systematic reviews and Meta-Analyses (PRISMA) checklist.^16^

A realist review method was compared to acceptable standards and compared to accepted standards of systematic review reporting (See Appendix*^14–16^).

### Step1. Clarify the scope of the review and key theories

A computerised search of PubMed, Google scholar, Springer link and Cochrane Library was conducted for the years 2000 to 2021, using search terms: Medium Secure Mental Health Unit, Secure Mental Health Facility, Secure Psychiatric Unit Australia. This initial scoping review was conducted to understand the heterogeneity and complexity of the area of interest and to refine the theories extant and to inform the inclusion/exclusion criteria.

This initial search identified 1570 unique articles but only one systematic review and meta-analysis.^8^

The studies were of variable methodologies and quality.

Several pertinent themes emerged from this scoping review including the impact of the social climate, progress during and after admission to secure units, risk reduction strategies in the units, physical health monitoring and wellbeing, characteristics and needs of patients in mediums secure settings.

An additional search using keywords including ‘forensic mental health legislation’ revealed policy documents, articles and editorials from Australian, UK and European sources. The information obtained from the initial scoping exercise provided the basis for a realist synthesis methodology and a further analysis of the research question.

### Step 2. Search for evidence

From the scoping review it was evident that the area was characterised by complexity, contextual specificity with variable quality of studies and sources of evidence. A subsequent electronic search was completed using four electronic databases; PubMed, SpringerLink, Cochrane library and Google search engine (*n* = 496) and yielded 85 papers after applying the inclusion criteria and the removal of duplicates.

The search terms ((‘medium secure unit*’ OR ‘high secure unit*’ OR ‘secure psychiatric unit’ OR ‘forensic mental health’ OR ‘secure psychiatric hospital’ OR ‘medium secure mental health rehabilitation unit*’ OR ‘secure mental health unit*’ OR ‘high secure hospital’ OR ‘medium secure hospital’ OR ‘therapeutic security’) OR (‘secure mental health unit*’)) AND (outcome* OR recover* OR rehabilitation).

### Step 3. Appraise primary studies and extract data

An in-depth review of the 18 papers conducted by two of the authors revealed three data categories of relevance to our aims:

a. Studies focussed on length of stay in medium secure units.
b. Studies focussed on patient characteristics associated with outcomes.
c. Studies focussed outcomes following discharge.

### Step 4: Data extraction, analysis, and synthesis

#### Inclusion/exclusion criteria

The articles found were reviewed according to inclusion criteria of:

a. Published in English
b. Published between 2000 and 2021
c. Key word/topic/theme

The articles excluded were as follows:

a. Non-English publication
b. Single case studies
c. Studies of forensic inpatient services not specifying medium secure
d. Studies with the primary aim is evaluating outcome measures
e. Papers reporting on regional legislation

**Figure 1.**
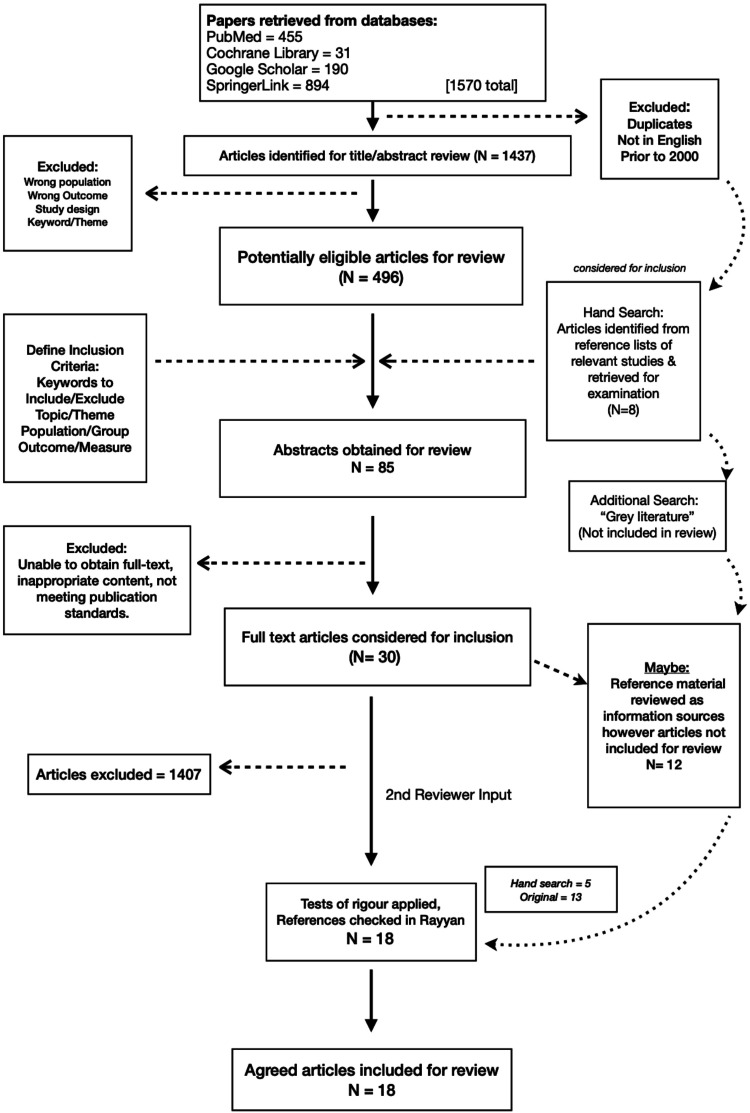
Flow diagram of literature search.

An additional eight potential articles were found in the hand search, of which a further five were included for the final literature review (Figure 1).

## Results

### Study quality

12 of these studies were retrospective cohort study designs.

The largest of these studies^9,17^ were from within the UK and covered periods of 8 and 10 years, respectively. The articles examined are summarised in Table 2.

**Table 2.** Summary of articles

Author/date/Place	Title	Population	*N* =	Key findings	Study type
**Earnshaw et al., 2019 England**	A retrospective study comparing the length of admission of medium secure unit patients admitted in the three decades since 1985	Admission to secure hospitals	169	Increased LOS* in study period	Retrospective cohort study (1985–2012)
**Long et al., 2015 England & Wales**	Treatment progress in medium security hospital settings for women: Changes in symptoms, personality and service need from admission to discharge: Women’s progress in medium security hospitals	Women admitted to medium secure	70	Improvement clinically. Symptoms, personality, and reduction in unmet needs	Single cohort pre/post design (2002–2009)
**Sharma et al., 2015 London**	The virtual institution: Cross-sectional length of stay in general adult and forensic psychiatry beds	Census of psychiatric bed use	1085	LOS* increased with increasing security of setting	Cross-sectional survey
				‘New long-stay’ patients increasing despite community-based care. Impact on acute admission beds. Use of hospital beds can be modelled using phases of care (short, medium, and long term) Need for population based prospective observational studies of care pathways to inform mental health and criminal justice and commissioning practices and policies. Need for standardise assessments of need for therapeutic security and readiness to step down into less secure place	
**Kasmi et al., 2020 England**	A comparison of long-term medium secure patients within NHS and private and charitable sector units in England	Long-stay patients in medium secure	285	178 NHS & 107 PCS patients included with mean LOS* similar: 163 & 164 months	Cohort administrative data
**Duke et al., 2018 England**	Long-stay in forensic psychiatric care in the UK. Social psychiatry and psychiatric epidemiology	Census data	2287	18.1% long-stay in medium secure with wide range 0%–50%. MHA section, admission source and current ward type influenced LOS*	Cross-sectional of administrative data
**Davies et al., 2007 UK**	Long-term outcomes after discharge from medium secure care: a cause for concern	Patients discharged from medium secure	550	On discharge risk of death including by suicide & reoffending	Retrospective cohort study
**Tabita et al., 2012 Sweden**	Criminal recidivism and mortality among patients discharged from a forensic medium secure hospital	Discharges from medium secure	88	On discharge risk of death including by suicide & reoffending	Retrospective cohort study
**Coid et al., 2007 England & Wales**	Comparison of outcomes following after-care from forensic and general adult psychiatric services	Patients discharged from medium secure	1613	Gender, younger age, early onset of offending, previous convictions, comorbid or primary diagnosis of personality disorder predicted offending	Retrospective cohort study of reconvictions & risk factors
**Maden et al., 2004 England & Wales**	Offending in psychiatric patients after discharge from medium secure units: Prospective national cohort study	Patients offending after discharge from medium secure units	145	Strongest predictor of reoffending was previous offending	Retrospective cohort (1997–1998) study of reconvictions & risk factors
**Tully et al., 2019 London**	Follow-up study of 6.5 years of admissions to a UK female medium secure forensic psychiatry unit	Female patients in medium secure forensic units	50	Improvement in clinical & risk assessments. Longer duration of admission associated with reduced reoffending but not readmission	Retrospective cohort study (2008–2014)
**Riberiro et al., 2015 London**	Clinical characteristics and outcomes on discharge of women admitted to a medium secure unit over a 4-year period	Female patients discharged from medium secure	45?	High proportion of ethnic minorities. Significantly reductions in self-harm and risk as measured by the HCR-20	Prospective cohort study (2008–2012)
**Gibbon et al., 2012 Leicester**	The influence of admission characteristics on outcome: Evidence from a medium secure forensic cohort	Admissions to a single medium secure unit	550	Increased severity of risk over time with a reduced time to reoffending on discharge. Outcomes related to being young, increased number of prior offences, the gravity of prior offences and legal designation of psychopathic disorder)	Retrospective cohort study (1983–2003)
**Coid et al., 2001 England & Wales**	Predicting admission rates to secure forensic psychiatry services	Admissions to high & medium secure	3155	Admission rates have a linear correlation with measures od socioeconomic deprivation	Retrospective cohort study (1988–1994)
**Gosek et al., 2021 Poland**	Treatment resistance and prolonged length of stay among schizophrenia inpatients in forensic institutions	Patients with schizophrenia in medium secure unit	87	LOS* influenced by family history of mental illness, substance dependence, severity of offences, treatment resistance and current treatment using more than 1 antipsychotic	Retrospective cross-sectional study (2014–2018)
**Shah et al., 2011 London**	Factors associated with length of admission at a medium secure forensic psychiatric unit	Patients discharged from a male medium secure ward	295	LOS*>2 years associated with diagnosis of psychotic disorder, multiple admissions, detention under an order, being on a restricted order and a history of moderate violent offending	Retrospective cross-sectional study (1999–2008)
**Gosek et al., 2020 Warsaw**	Factors influencing length of stay of forensic patients: Impact of clinical and Psychosocial variables in medium secure setting	Patients in a medium secure unit	150	LOS* influenced by duration of mental illness, severity of index offence, offending while psychotic, if offending occurred over a period of time, persistent psychosis, multiple antipsychotics and a diagnosis of schizophrenia or schizoaffective disorder	Retrospective cross-sectional study (2014–2018)
**Vollm et al., 2018 England**	Characteristics and pathways of long-stay patients in high and medium secure settings in England; A Secondary publication from a large mixed-Methods study. Front. Psychiatry 2018	Patients in high & medium secure units	401	Long-stay defined as 5 years in medium secure, 10 years in high secure, 15 combined. Long-stay group demonstrated similarities across both high & medium secure settings. Suggest separate category/provision of care	Retrospective cohort study (2013)
**Davoren et al., 2015 Ireland**	Factors affecting length of stay in forensic hospital setting: Need for therapeutic security and course of admission	Admission to medium secure forensic hospital	279	Diagnosis at preadmission predicted legal status	Naturalist prospective cohort study January 2010-30 June 2014
				Dundrum scales 1 &2 predicted length of stay	

Whilst each paper looked at several elements impacting medium secure forensic patients, it became apparent that admissions, discharges and prolonged stays were multi-factorial.

Some of the studies focussed on length of stay as the main variable of interest.^3,5–7,17–19^

Several studies examined admission characteristics of patients^20–22^ four studies looked at characteristics of people within medium secure^23,24,26^ and four primarily^27^ analysed post discharge outcomes.^26,28–30^

Though most of the chosen articles featured several key areas of interest, they were broadly categorised into articles with a primary focus on length of stay, admission characteristics or outcomes.

The ideal upper limit for length of admission to medium secure units (MSUs) has been defined as 2 years.^2^

Prolonged admissions were more likely to be associated with diagnosed psychotic disorders, multiple prior admissions, detainment under hospital, or restriction orders or history of moderately violent offending.

Findings are briefly summarised in Table 3.

**Table 3.** Summary of results

Length of Stay
Shah et al. (2011)^7^ report that prolonged admissions were more likely to be associated with diagnosed psychotic disorders, multiple prior admissions, detainment under hospital or restriction orders or history of moderately violent offending. They concluded that service reconfiguration was required in order to offer lower level security rehabilitation
Gosek et al. (2020)^5^ found a significant gap in available data for Eastern or Central European patients & set out to identify potential variables that may determine length of stay. These included duration of mental illness; severity of index offence; whether the crime was committed due to psychotic symptoms or during treatment discontinuation; if the index offences were crimes committed over an extended period of time; persistent psychosis; multiple psychotropic treatments; diagnosis of schizophrenia & schizoaffective disorder
From a database of 63 variables, they found that length of stay was influenced by duration of mental illness; severity of index offence; whether the crime was committed due to psychotic symptoms or during treatment discontinuation. They also identified external influencing factors such as unique characteristics of each European nations judicial system, admission criteria, general psychiatric resources and supports^5^
Sharma et al. (2015)^3^ note that the LOS in psychiatric hospitals is of interest in health service planning. Accordingly, their study examined whether patients were placed according to specialist need or related to cross-sectional length of stay
It was found that LOS increased with the level of therapeutic security, with notably longer stays for those under forensic detention
In 2018, Duke et al.^17^ commented on the ethical & financial issues that arise from unnecessarily long-stays in secure care environments. They examined 3 high secure units & 23 medium secure units in a cross-sectional study of forensic facilities in England. They aimed to identify the prevalence of long-stays. They found that admission sources, ward type & mental health Act status were independent variables related to long-stays
Imposing a loss of privacy, repetition in daily routine & low stimuli; the secure care facility can be extremely restrictive
In addition, they commented on the concerning tendency for patients to be moved from the same or lower levels of security, noting the suggestion of a ‘*trajectory of movement within the system rather than progression outwards*’ (Duke et al., 2018)^17^
Kasmi et al. (2020)^6^ examined forensic psychiatric services in England, comparing characteristics of long-stay patients in both private & charitable sector units (PCS) & national health service (NHS)
It was found that the PCS had a high prevalence of people with challenging, including assaultative behaviours it was reported that NHS long-stay patients were more ethnically diverse. The authors considered that the higher proportion of Black & ethnic minority groups detained under mental health legislation in the UK warranted further deliberation & conceded that diagnosis of serious mental illness may have impacted the overrepresentation noted in their sample
Earnshaw et al. (2019)^18^ compared admission data from an MSU over three decades, noting an increase in median admission length from 167 days in 1985 to 580 days in 2012. There was a significant increase in the length of admissions & a noted decrease in the amount of admissions to this facility. The study included 179 patient records, observing trends in diagnosis, length of stay & discharge destination. It was found that fewer patients were directly discharged to home addresses, with many being sent to long-term MSU’s, lower security psychiatric units or supported accomodations
**Admission factors**
Given the scarcity of resources, being able to identify & predict the length of stay at the time of admission to a forensic unit can be advantageous
A study predicting the length of stay in medium secure settings utilised the DUNDRUM-1 triage security scale & DUNDRUM-2 triage urgency scale (Davoren, 2015).^22^ This prospective study aimed to estimate the length of stay at the time of admission
These measures were used to assess the need for therapeutic security & urgency, along with the HCR-20 to assess for risk of violence. The cohort of 279 patients were admitted between 1st January 2010 & 30th June 2014 & followed up until 1st July 2015^22^
All admissions to a medium secure mental health hospital in Ireland were collated over a period of 54 months and followed up for a total of 66 months
Male gender predicted a longer length of stay. A shorter length of stay was predicted when there was no mental disorder other than adjustment disorder
Other diagnoses (schizophrenia spectrum disorders, affective disorders, organic disorders) had no bearing on length of stay
In a separate study, Coid et al. (2001b)^9^ conducted a retrospective review of admissions to secure forensic units in 14 regional authorities in England and Wales for the years 1988–94. The admission rates increased in areas of social deprivation, ethnicity and availability of low secure beds in the region^9^
Gibbon (2012)^21^ set out to examine whether patient admission characteristics were becoming increasingly severe, whether the discharge outcomes were notably deteriorating & whether an increased severity at admission correlated with a worse outcome
They identified 18 factors which may potentially predict reconviction & determined that 17 of these could be regarded as admission characteristics. The remaining factor was determined to be length of stay. Over 20 years, 264 of the 542 patients followed up were re-convicted of an offence. This was 48.7% of the cohort
The authors recognise that changes to service structures likely represent the increased concentration of high-risk patients in medium security with the need to provide service to prison. In addition they noted amongst those admitted an increase in reported childhood behavioural disturbance & historical abuse & problematic school interactions. There was also a noted increase in substance misuse & self-harm
**Patient characteristics**
There is limited data on women in medium secure facilities. Ribeiro et al., (2015)^24^ studied the characteristics of 45 admissions to the female ward of an (MSU) in the UK over a period of 4 years. Demographic data, clinical outcomes & HoNOS-secure, HoNOS & HCR-20 assessments were collected. ICD-10 diagnosis & WHO-QoL-BREF surveys were utilised. It was found that within this patient cohort there was a high proportion of ethnic minorities, high rates of childhood & adult abuse & low socioeconomic status. 62.2% were diagnosed with schizophrenia & 57.8% had multiple diagnoses
In 2015, Long et al. examined a female cohort in an MSU. They reported a significant reduction in risk behaviour which was paralleled by increases in treatment engagement
Tully et al. (2019)^31^ noted examined 50 admissions to a female medium secure unit between April 2008 & November 2014 in London, UK. They obtained demographic, clinical risk assessment & quality of life data using HCR-20 & HoNOS-secure. Patients significant histories of physical or sexual abuse or neglect present in 52% & 44% of their population reported a history of self-harm. 46% had alcohol &/or drug misuse histories
They found that results indicated that longer admission duration & restrictions imposed upon discharge were associated with reduced rates of reoffending, whilst readmission rates were not affected
Vollm et al. (2018)^25^ found that personality pathology & risk of staff assault impacted treatment. 60% of patients were diagnosed with schizophrenia, 47% personality disorder (predominantly antisocial & borderline), 16% diagnosed with intellectual disability. 7% of the long-stay sample had not been convicted of any offence & 16.5% had no index offence. For 33%, an unsuccessful attempt to a lower security setting was recorded over the preceding 5 years (Völlm et al., 2018)^25^
**Post discharge outcomes**
Studies examining outcomes following a stay in medium secure have similarly examined clinical, functional, and criminal domains
The largest of these studies (Coid et al., 2007)^32^ focussed on reconvictions and found that gender, younger age, early onset of offending, previous convictions a diagnosis of personality disorder predicted offending in a retrospective cohort of 1613 patients discharged from medium secure
Maden et al. (2004)^28^ in a smaller study of 145 patients found the strongest predictor of reoffending was previous offending
Davies et al. (2007)^26^ looked into describing mortality, rates of reconviction, violent behaviour, readmission & employment following discharge from medium secure units. They studied the first admission cohort of a medium secure service over a 20 year period
The results suggested that the overall long-term outcome for this cohort of patients was poor. They experienced high rates of mortality, reconviction, readmission & few of them gained employment
Tabita et al. (2012)^29^ reported a rate of 38% recidivism in their study population who were sentenced to forensic psychiatric treatment & discharged between 1992 and 2007

### Discussion

Specialised secure mental health services have been set up with a dual purpose of optimising mental health care for patients who have not had their needs met in less restrictive care while also attending to community risk issues. There are numerous research challenges in these settings, which are reflected in the predominance of retrospective cohort studies using administrative data.

Völlm et al. (2018)^25^ highlight the potential ethical implications of prolonged admissions. Some people admitted to forensic psychiatric care may be held for longer than they had been incarcerated for the same offence if not mentally disordered.

#### Length of stay

Six of the studies reviewed examined factors in length of stay (LOS). The evidence is mixed. Despite the ethical concerns of unnecessary long LOS often influenced by limited step-down options, there are also individuals where a longer than recommend 2 years LOS is required for optimal outcomes.^26^ This issue is identified in smaller studies in this review examining issues for females in medium secure.^23,31^ There are other factors that are beginning to be a focus of research including the impact of ethnicity,^24^ and socioeconomic variables on LOS.^9^

In the studies reviewed, extended LOS were related to several common factors including multiple prior admissions; history of offending, particularly recidivism and severe offending; diagnosis of psychotic disorder (especially treatment resistant) and referrals from higher secure settings.

The rationale for understanding patient characteristics is to be able to predict the care needs and potential length of stay. As this patient population requires a high level of support and allocation of funding, there are often fiscal restrictions and limited resources available.

Most of the available literature pertaining to patient characteristics is published in the UK or Europe, with definitions of standards of care and quantification of risk often being led by clinicians and investigators from this part of the world.^3,5–9,21,23,24,26,28–30^

Earnshaw et al.^18^ noted the proportion of schizophrenia diagnoses amongst their study population and the inherent risks involved when re-integrating patients into the community. They examined 47, 65, 37 and 30 patients who were admitted to the MSU over the years 1985, 1995, 2005 and 2015. Most were diagnosed with paranoid schizophrenia. The authors commented that offering extended care in supported environments may be beneficial in reducing risk to others at a population level (2019).^18^

Sharma et al. (2015) noted that when studying systems, general adult and forensic psychiatric beds are preferentially compared as they tend to be inter-dependent.^3^

Duke et al. (2018) concluded that there was a lack of consistency in admission criteria and discharge procedures. They also noted that sociodemographic factors may hold less significance in prediction of long-stays in forensic settings.

In 2020, Kasmi et al. found that private and charitable sector units (PCS) had a higher likelihood of accepting care of civil detention cases and intellectual disabilities, who were often long-stay residents in secure services.

Interestingly, they note similar difficulties with the development of medium secure hospital services (2020).

Gosek et al. (2021) reported that increased lengths of stay were noted in cases of: index offences of crimes committed over an extended period; persistent psychosis; multiple psychotropic treatments; diagnosis of schizophrenia and schizoaffective disorder. This review of 150 patients in medium secure setting also analysed psychosocial variables associated with length of stay.^5^

#### Admission factors

Coid et al. (2001a^20^) highlight the need to be aware of the social determinants of mental health, especially in health service planning. Factors associated with increased LOS (younger age, history of offending, males, diagnosis of personality disorder) also influenced admission to medium secure.

Personality pathology and risk of staff and co-patient assault further impact upon treatment and management. Poor response to treatment, ongoing safety concerns and lack of suitable step-down facilities are also identified as reasons for delayed discharge.

The findings from Davoren (2015)^22^ revealed that approximating the length of stay for forensic patients may be useful for clinicians, service planners and commissioners, considering the financial implications of secure psychiatric care.^22^ They reported that legal status upon admission and predicted legal status was indicative of length of stay when criminal justice disposal predicted shorter length of stay (Davoren, 2015).^22^

Gibbon 2012, noted increased severity in admission characteristics that was deemed to be anecdotal and this study supported this observation.

#### Patient characteristics

Attributes of long-stay forensic patients in high and medium secure settings in England remain poorly understood. Völlm et al. (2018)^25^ sought to clarify the patient characteristics that led to admissions beyond 5 years in medium secure care. In their sample of 401 forensic patients who met the criteria for long-stay it was determined that one fifth of the forensic inpatient population were represented by long-stayers.

It was found that of the sample of patients meeting criteria for long-stay, the authors found that 58% of these were violent offenders (22% both sexual and violent), 27% had committed violent or sexual offences in an institutional setting and 26% had committed a serious assault on staff in the preceding 5 years (Völlm et al., 2018).^25^

#### Discharge outcomes

Particularly sobering are the studies identifying the risks of suicide and death on discharge from medium secure.^8,25,28^

Clinicians need to be aware that discharge from medium secure facilities can be a time of high risk of suicide.

As MSUs have an equally important role in improving the mental health of patients it is of concern that both Davies et al. (2007)^26^ in a study of 550 patients and Tabita et al. (2012)^29^ in a study of 88 patients noted an increased risk of death as well as reoffending.

### Strengths and limitations

This review was conducted with the aim of understanding why there is a difficulty in accessing secure care. We found that there was limited local data pertaining to our area of interest. It was interesting to note that a number of international researchers considered a number of different variables which may impact an individual patient experience as well as service provision and resource limitations.

We considered this to be part of a more in-depth study to begin to understand how to inform policy around the provision of a new MSU.

Whilst a systematic review remains a gold standard of literature review, it does not address how or if an intervention works and can often result in some aspects being left unresolved. A realist review was determined to be an appropriate investigation tool; however, this remains a new and emerging method.^13^

Our research team were English speakers only and our review included some limitations.

Non-English documents were excluded from our searches. An inclusive approach and broad definitions to search terms were applied.

There are several challenges influencing the type and quality of research in secure settings which is reflected in the predominance of retrospective studies using administrative data identified in this review. In addition, these services vary based on country and jurisdictions, with most studies in this review emanating from the United Kingdom.

We conducted our realist review within a 1 year timeframe. Subsequently, it became difficult to publish the results of the analysis.

The review did not look at details of the programs run in MSUs and the influence of care offered on outcomes. Many studies were from the UK which needs to be considered in extrapolating to other jurisdictions. MSUs in the UK are managed by forensic services and admit patients with serious violent or sexual offences (that would meet the criteria for high secure admission in Queensland, Australia).

In Queensland, the secure rehabilitation units are generally managed by district services. The UK also has ‘secure rehabilitation units’ analogous to those in Queensland and managed by districts (not forensic services).

Ultimately it was found that the value of the realist synthesis is to provide more contextual information in the development of program theory.

### Future research directions

The authors plan to use this information to inform medium care pathways in the Australian context.

There is need for further research to test theories developed from this review.^32^ These include a need for improved understanding of the medium secure MOS; incorporating complex care reviews in management/treatment planning; and discharge planning to ensure transitional care.

A retrospective cohort study of patients currently admitted to The Park Centre for Mental Health’s Medium Secure Unit has been considered.

In addition, a prospective study will be undertaken to do a longitudinal assessment of referrals to new medium secure rehabilitation unit. The objectives would be to evaluate the policy and procedures developed for admission to that facility, the degree of integration with the broader mental health system and the discharge options available.

## Conclusion

This study was undertaken to address the need for development of a referral pathway into local medium secure services. A realist review was utilised with the aim of synthesising relevant data and applying it in the appropriate context to inform policy and procedure.

MSUs are necessary to meet the specialised needs of a minority of patients with mental illness requiring secure mental health care to recover. They need to be well integrated into the broader mainstream mental health service that will refer to the unit and be part of care after discharge.

Pathways of care need to be clear to ensure less restrictive interventions have been tried prior to referral to a medium secure and that step-down options are available that give a level of support to mitigate against the risk of suicide and reoffending.

## Supplemental Material

Supplemental Material - Factors associated with length of stay in medium secure units: A realist review Supplemental Material for Factors associated with length of stay in medium secure units: A realist review by Wajeeha Zagham, Steve Kiseley, Terry Stedman, Karen Brown, and Frances Dark in Australasian Psychiatry

## Data Availability

On request to the corresponding author.*
