# Supplementary material for: Factors associated with length of stay in medium secure units: A realist review
Source: Australas Psychiatry. 2024 Sep 24;32(6):531–41. doi: 10.1177/10398562241286627 (PMC11605979; doi:10.1177/10398562241286627)
Supplement: Supplemental Material - Factors associated with length of stay in medium secure units: A realist review [file sj-pdf-1-apy-10.1177_10398562241286627.pdf]

Appendix I - *RAMESES Publication Standards Checklist*

	Yes n (%)	No n (%)	Somewhat/Vaguely n (%)	
TITLE				**Comments**
1. In the title, identify the document as a realist synthesis or review	Y			
ABSTRACT				
2. Contain brief details of: background, review question/objectives, search strategy, methods of selection, appraisal, analysis, and synthesis of sources, main results, implications for practice	Y			
INTRODUCTION				
3. Explain why the review is needed and what it is likely to contribute to existing understanding of the topic area	Y			
4. Objectives and focus of review: a) State the objective(s) of the review and/or the review question(s)	Y			
4. Objectives and focus of review: b) Define and provide a rationale for the focus of the review	Y			
METHOD				
5. Changes: Any changes made to the review process that was initially planned should be briefly described and justified	Y			
6. Rationale: Explain why realist synthesis was considered the most appropriate method to use	Y			
7. Scoping the literature: Describe and justify the initial process of exploratory scoping of the literature	Y			
8. Searching process: State and provide a rationale for how the iterative searching was done. Provide details on all the sources accessed for information in the review	Y			
9. Selection and appraisal of documents: Explain how judgments were made about including and excluding data from documents, and justify these	Y			Combined PRISMA & RAMSES = increased rigour
10. Data extraction: Describe and explain which data or information were extracted from the included documents and justify this selection	Y			
11. Analysis and synthesis process: (a) Describe the analysis process in detail. This section should include information on the constructs analyzed and describe the analytic process	Y			
11. Analysis and synthesis process: (b) Describe the synthesis processes in detail	Y			
RESULTS				
12. Document flow diagram: Provide details on the number of documents assessed for eligibility and included in the review with reasons for exclusion at each stage as well as an indication of their source of origin	Y			
13. Document characteristics: Provide information on the characteristics of the documents included in the review	Y			
14. Main findings: Present the key findings with a specific focus on theory building and testing	Y			
DISCUSSION				

15. Summary of findings: Summarize the main findings, taking into account the review's objective(s), research question(s), focus, and intended audience(s)	Y			
16. Strengths, limitations, and future research directions: a) Discuss the strengths and limitations of the review	Y			
17. Comparison with existing literature: Where applicable, compare and contrast the review's findings with the existing literature on the same topic	Y			

Wong, G., Greenhalgh, T., Westhorp, G. et al. RAMESES publication standards: realist syntheses. BMC Med 11, 21 (2013). https://doi.org/10.1186/1741- 7015-11-21[14]

Appendix II

STROBE Statement—checklist of items that should be included in reports of observational studies

	Item No.	Recommendation	Page No.	Relevant text from manuscript
**Title and abstract**	1	(*a*) Indicate the study's design with a commonly used term in the title or the abstract		
		(*b*) Provide in the abstract an informative and balanced summary of what was done and what was found		
**Introduction**				
Background/ rationale	2	Explain the scientific background and rationale for the investigation being reported		
Objectives	3	State specific objectives, including any prespecified hypotheses		
**Methods**				
Study design	4	Present key elements of study design early in the paper		
Setting	5	Describe the setting, locations, and relevant dates, including periods of recruitment, exposure, follow-up, and data collection		
Participants	6	(*a*) *Cohort study*—Give the eligibility criteria, and the sources and methods of selection of participants. Describe methods of follow-up<br>*Case-control study*—Give the eligibility criteria, and the sources and methods of case ascertainment and control selection. Give the rationale for the choice of cases and controls<br>*Cross-sectional study*—Give the eligibility criteria, and the sources and methods of selection of participants		
		(*b*) *Cohort study*—For matched studies, give matching criteria and number of exposed and unexposed<br>*Case-control study*—For matched studies, give matching criteria and the number of controls per case		

Variables	7	Clearly define all outcomes, exposures, predictors, potential confounders, and effect modifiers. Give diagnostic criteria, if applicable
Data sources/ measurement	8*	For each variable of interest, give sources of data and details of methods of assessment (measurement). Describe comparability of assessment methods if there is more than one group
Bias	9	Describe any efforts to address potential sources of bias
Study size	10	Explain how the study size was arrived at

Continued on next page

Quantitative variables	11	Explain how quantitative variables were handled in the analyses. If applicable, describe which groupings were chosen and why
Statistical methods	12	(*a*) Describe all statistical methods, including those used to control for confounding
		(*b*) Describe any methods used to examine subgroups and interactions
		(*c*) Explain how missing data were addressed
		(*d*) *Cohort study*—If applicable, explain how loss to follow-up was addressed<br>*Case-control study*—If applicable, explain how matching of cases and controls was addressed<br>*Cross-sectional study*—If applicable, describe analytical methods taking account of sampling strategy
		(*e*) Describe any sensitivity analyses
**Results**		
Participants	13*	(a) Report numbers of individuals at each stage of study—eg numbers potentially eligible, examined for eligibility, confirmed eligible, included in the study, completing follow-up, and analysed
		(b) Give reasons for non-participation at each stage
		(c) Consider use of a flow diagram
Descriptive data	14*	(a) Give characteristics of study participants (eg demographic, clinical, social) and information on exposures and potential confounders
		(b) Indicate number of participants with missing data for each variable of interest
		(c) *Cohort study*—Summarise follow-up time (eg, average and total amount)
Outcome data	15*	*Cohort study*—Report numbers of outcome events or summary measures over time
		Case-control study—Report numbers in each exposure category, or summary measures of exposure
		Cross-sectional study—Report numbers of outcome events or summary measures
Main results	16	(*a*) Give unadjusted estimates and, if applicable, confounder-adjusted estimates and their precision (eg, 95% confidence interval). Make clear which confounders were adjusted for and why they were included
		(*b*) Report category boundaries when continuous variables were categorized
		(*c*) If relevant, consider translating estimates of relative risk into absolute risk for a meaningful time period

Continued on next page

Other analyses	17	Report other analyses done—eg analyses of subgroups and interactions, and sensitivity analyses
**Discussion**		
Key results	18	Summarise key results with reference to study objectives
Limitations	19	Discuss limitations of the study, taking into account sources of potential bias or imprecision. Discuss both direction and magnitude of any potential bias
Interpretation	20	Give a cautious overall interpretation of results considering objectives, limitations, multiplicity of analyses, results from similar studies, and other relevant evidence
Generalisability	21	Discuss the generalisability (external validity) of the study results
**Other information**		
Funding	22	Give the source of funding and the role of the funders for the present study and, if applicable, for the original study on which the present article is based

*Give information separately for cases and controls in case-control studies and, if applicable, for exposed and unexposed groups in cohort and cross-sectional studies.

Note: An Explanation and Elaboration article discusses each checklist item and gives methodological background and published examples of transparent reporting. The STROBE checklist is best used in conjunction with this article (freely available on the Web sites of PLoS Medicine at http://www.plosmedicine.org/, Annals of Internal Medicine at http://www.annals.org/, and Epidemiology at http://www.epidem.com/). Information on the STROBE Initiative is available at www.strobe-statement.org.

Appendix 2

von Elm E, Altman DG, Egger M, Pocock SJ, Gotzsche PC, Vandenbroucke JP. The Strengthening the Reporting of Observational Studies in Epidemiology (STROBE) Statement: guidelines for reporting observational studies. Lancet. 2007;370(9596):1453-1457. PMID: 18064739 [15]

Appendix III

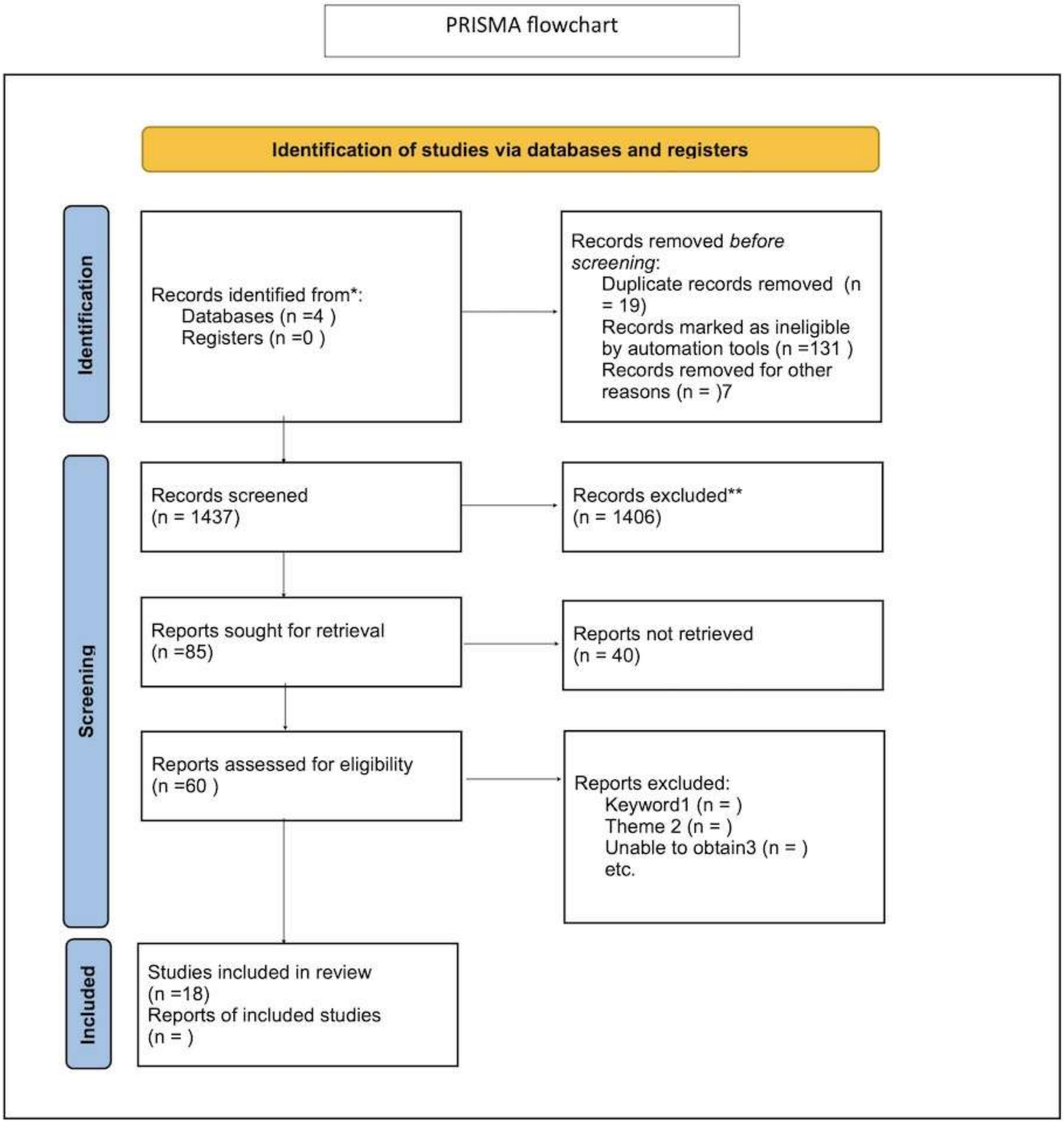

*Consider, if feasible to do so, reporting the number of records identified from each database or register searched (rather than the total number across all databases/registers).

**If automation tools were used, indicate how many records were excluded by a human and how many were excluded by automation tools

FIGURE 2

Page MJ, McKenzie JE, Bossuyt PM, Boutron I, Hoffmann TC, Mulrow CD, et al. The PRISMA 2020 statement: an updated guideline for reporting systematic reviews. BMJ 2021;372:n71. doi: 10.1136/bmj.n71 [16]
